# On Modelling Minimal Disease Activity

**DOI:** 10.1002/acr.22687

**Published:** 2016-02-23

**Authors:** Christopher H. Jackson, Li Su, Dafna D. Gladman, Vernon T. Farewell

**Affiliations:** ^1^ MRC Biostatistics Unit, Cambridge Institute of Public Health, Cambridge UniversityCambridgeUK; ^2^ University of Toronto and Toronto Western HospitalTorontoOntarioCanada

## Abstract

**Objective:**

To explore methods for statistical modelling of minimal disease activity (MDA) based on data from intermittent clinic visits.

**Methods:**

The analysis was based on a 2‐state model. Comparisons were made between analyses based on “complete case” data from visits at which MDA status was known, and the use of hidden model methodology that incorporated information from visits at which only some MDA defining criteria could be established. Analyses were based on an observational psoriatic arthritis cohort.

**Results:**

With data from 856 patients and 7,024 clinic visits, analysis was based on virtually all visits, although only 62.6% provided enough information to determine MDA status. Estimated mean times for an episode of MDA varied from 4.18 years to 3.10 years, with smaller estimates derived from the hidden 2‐state model analysis. Over a 10‐year period, the estimated expected times spent in MDA episodes of longer than 1 year was 3.90 to 4.22, and the probability of having such an MDA episode was estimated to be 0.85 to 0.91, with longer times and greater probabilities seen with the hidden 2‐state model analysis.

**Conclusion:**

A 2‐state model provides a useful framework for the analysis of MDA. Use of data from visits at which MDA status can not be determined provide more precision, and notable differences are seen in estimated quantities related to MDA episodes based on complete case and hidden 2‐state model analyses. The possibility of bias, as well as loss of precision, should be recognized when complete case analyses are used.

## INTRODUCTION

There is currently considerable interest in the concept of minimal disease activity (MDA) for rheumatic diseases. The Outcome Measures in Rheumatology Clinical Trials 6 Conference agreed on a conceptual definition of MDA as “that state of disease activity deemed a useful target of treatment by both the patient and physician, given current treatment possibilities and limitations.”

Box 1 **Significance & Innovations**

- The use of a “hidden” 2‐state model provides a useful framework for analysis of data on minimal disease activity (MDA).
- Inclusion of data from clinic visits at which only partial information is available regarding MDA status leads to increased precision of estimation and protection against bias.

Coates et al 1 examined a specific definition of MDA for psoriatic arthritis (PsA) originally suggested in Coates et al 2. They compared patients who achieved sustained MDA, defined as satisfying the definition for a minimum of 12 months, to those not achieving this goal. In addition, they examined models for the time to first achievement of sustained MDA. There are challenges to the analysis of events defined by prolonged observation of a condition 3 as is necessary for the analysis of sustained MDA; patients may go in and out of an MDA state, and the time with MDA may be of interest. In this study we explore the use of a 2‐state model for the presence and absence of MDA in PsA. We also illustrate how the model may be used to quantify aspects of sustained MDA. Estimation of the model is based on clinical cohort data with patients being observed at intermittent time points, i.e., clinic visits. Estimation of the 2‐state model that incorporates visits with partial information on MDA status may be an improvement on estimation of the model based on visits with complete information only.

## PATIENTS AND METHODS

Our data consist of 7,024 clinical visits from 856 patients seen at the University of Toronto PsA Clinic since 2003. Patients were evaluated using a standard protocol every 6 to 12 months. Clinical assessments included a 68‐joint tender joint count (TJC), a 66‐joint swollen joint count (SJC), the Spondylarthritis Research Consortium of Canada enthesitis instrument (since 2008, and examination of Achilles tendons and plantar fascia insertions previously), and dactylitis measures using the Leeds Dactylitis instrument 4, 5. Skin assessment included both the body surface area (BSA) and the Psoriasis Area and Severity Index (PASI) 6. A clinically damaged joint count was recorded in addition to the TJC and SJC at each visit. Damaged joints were defined as those that had a reduced range of motion >20% of the range that could not be explained by joint effusion, joints that had undergone surgery, or joints showing deformity, subluxation, loosening, or ankylosis. The reliability of this measure has been demonstrated in the Toronto clinic and across Canada 7, 8. A physician global assessment was completed, and patients completed self‐reported questionnaires, including the Health Assessment Questionnaire (HAQ) and patient global assessments routinely. The criteria for the definition of MDA used by Coates et al 1 were ≥5 of the following 7 criteria: 1) TJC ≤1, 2) SJC ≤1, 3) PASI score ≤1 or BSA ≤3%, 4) patient pain visual analog score ≤15 mm, 5) patient global disease activity visual analog score ≤20 mm, 6) HAQ score ≤0.5, and 7) entheseal points ≤1.

**Figure 1:**
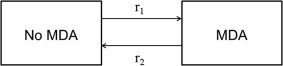
A 2‐state model for minimal disease activity (MDA).

The underlying basis of our proposed analyses is the 2‐state model illustrated in Figure 1. The model is based on the assumptions that a patient either has or does not have MDA and that, although patients are observed intermittently at clinic visits, a patient may change “state” at any point in time. Some MDA episodes may be sustained for 12 months or more, and some may be transient, lasting less than 12 months; these can be distinguished when simulating from the model. The parameters to be estimated from this model are the 2 rates of transition: r_1_ from no MDA to MDA and r_2_ from MDA to no MDA. Maximum likelihood estimation of the rates is used. If *i* and *j* are used to represent model states, where *i* and *j* take on values 1 or 2 depending on whether they represent the no MDA or MDA state, the maximum likelihood estimation only requires the specification of expressions for the probability of observing a patient in state *i* at one visit where MDA status can be determined and state *j* at the next visit where MDA status can be determined. This approach allows taking account of the intermittent observation of the patients and the length of time between any 2 clinic visits. For simplicity, r_1_ and r_2_ are taken to be constant over followup time, although this restriction can be relaxed.

From any particular clinic visit, information may be available on all, none, or a subset of the 7 binary criteria used to define MDA. Typically, estimation of the 2‐state model in Figure 1 would be based only on visits where either at least 5 of 7 criteria were observed and satisfied or at least 3 of 7 criteria were observed and not satisfied so that MDA/no MDA status can be determined. This is termed a complete case analysis. Two problems may arise with this approach. The first is that for some visits there may be information on some of the 7 MDA criteria, and if this information can be used to give some information on the likely MDA state at those visits then estimation of model parameters may be more precise. The second is that when only certain variables are available, this may, in some way, be informative about the likely MDA status. If this is the case, then basing information only on visits with a known MDA status may lead to biased estimation.

One approach to dealing with these 2 problems is to regard the 2‐state MDA model as a partially hidden multistate model. This means that while for some clinic visits we know MDA status, for others it is unknown or “hidden.” To fit a hidden Markov model, we need to define statistical models for the distributions of the 7 MDA defining criteria conditional on having MDA and conditional on not having MDA. Let X_1_, X_2_, … X_7_ represent the 7 binary variables that are needed to specify the 7 MDA criteria. In the definition of MDA, these are all binary indicators taking on values of 0 or 1, depending on whether a certain condition is satisfied. Models for these binary indicators will specify the distribution of each X_i_ conditional on MDA, f(X_i_ | MDA) and conditional on no MDA, f(X_i_ | no MDA), as Bernoulli random variables. The assumption is made that these variables are independently distributed, after conditioning on MDA status. Clearly without this conditioning on active disease status the independence assumption would be unreasonable, but we feel it is less problematic given the conditioning, although it is not likely to be exactly true.

While MDA is defined using binary indicators, an alternative approach to fitting the hidden multistate model is to directly model each of the variables used to define the criteria. This will require modelling 8 quantitative variables, as either PASI or BSA can be considered for the third criterion. For a clinic visit where MDA status is unknown, let x_all_ represent either the subset of the 7 binary indicators or the subset of the 8 variables used to define the binary indicators that are observed. Then instead of just the probability of being in the MDA or no MDA states being used for maximum likelihood estimation, the joint probability of being in a state and having the observed x_all_ becomes the basis of the likelihood estimation. When the state is unknown for a patient, the probability used for that clinic visit for estimation is just the probability function for the observed x_all_, f(x_all_) = f(x_all_ | MDA) Prob (MDA) + f(x_all_ | no MDA) Prob (no MDA). Prob (MDA) and Prob (no MDA) are calculated based on the multistate process for moving between the underlying states. The rates of moving between these states, and the probabilities of the binary outcome variables conditionally on the MDA states, can be examined simultaneously by maximum likelihood estimation.

When modelling the 8 quantitative variables, the integer patient pain and patient global activity scores are modelled as binomial variables taking integer values from 0 to 10, and the remaining 6 variables, which also take integer values (after multiplying HAQ and PASI by 100), are modelled as negative binomial variables. Alternative modelling could be considered, but these are adopted as convenient and reasonable approximations.

Based on estimation of the 2 rates, r_1_ and r_2_, it is also possible to estimate the expected time spent in the MDA state over any fixed time period, the average (mean) time that a patient remains in the MDA and no MDA states (mean sojourn time), and the probability of MDA occurring during a fixed time period, given the patient is not in the MDA state at the start of the time period. Furthermore, it is also possible to examine, via numerical evaluation or via simulation, comparable related measures associated with sustained MDA of at least 1 year's duration.

It is also possible to estimate the parameters of the hidden multistate model in Figure 1 using only the additional information from a single X variable. While not recommended in practice, this will also be done for illustrative purposes. Estimation for the various multistate models was done with the *msm* package (version 1.5.2) 9 in the statistical computing environment R 10.

## RESULTS

**Figure 2:**
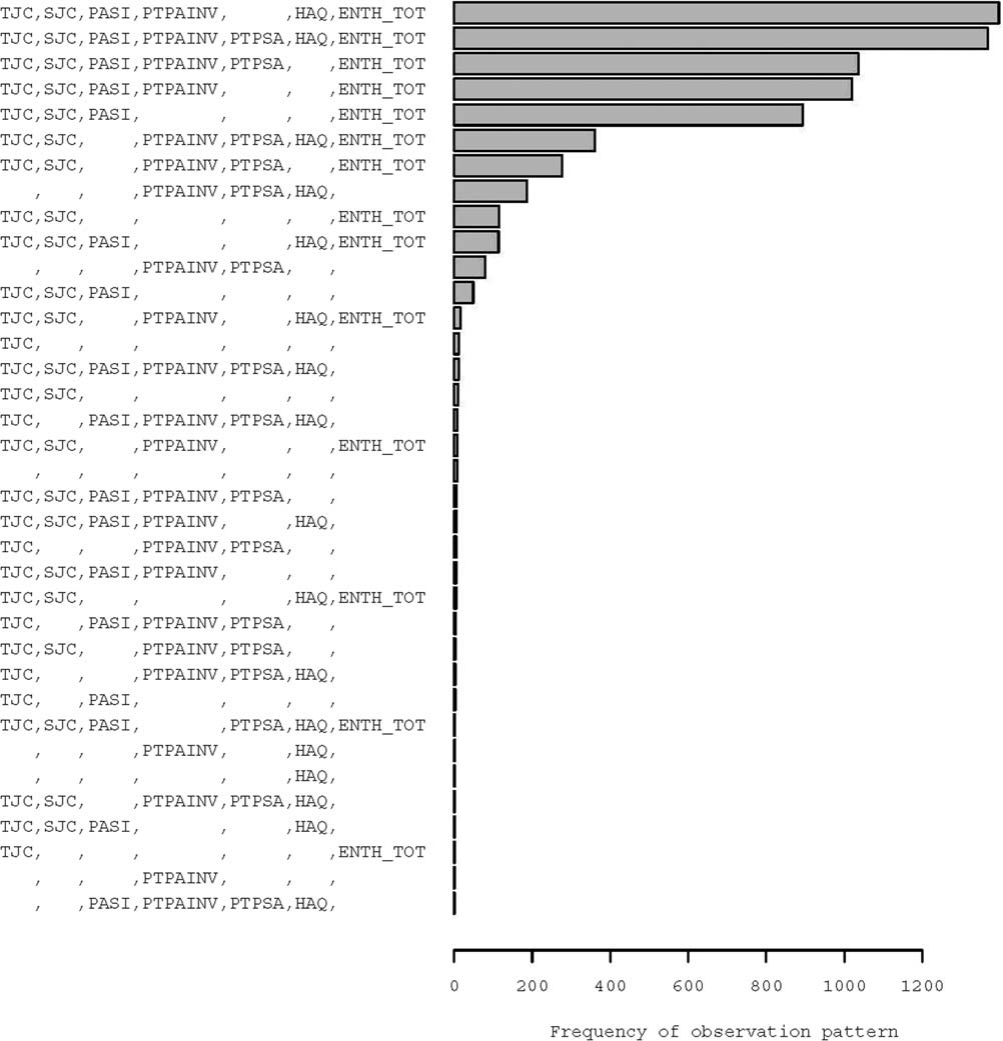
Pattern of missingness in binary indicators. TJC = total joint count; SJC = swollen joint count; PASI = Psoriasis Area and Severity Index; PTPAINV = patient pain visual analog score; HAQ = Health Assessment Questionnaire; ENTH TOT = entheseal points; PTPSA = patient global activity visual analog score.

Only 62.6% of 7,024 clinic visits had sufficient data to determine MDA status. However, all but 8 of these visits provided information on at least 1 of the MDA criteria. For example, the joint counts and enthesitis information were all recorded in approximately 95% of the visits. The number of visits with none, 1, 2, 3, 4, 5, 6, and 7 binary variables missing were 1,367, 2,807, 1,449, 924, 357, 96, 16, and 8, respectively. The pattern of missing data is displayed in Figure 2.

In our data set, 619 patients had more than 1 observation of confirmed MDA or no MDA. For all pairs of visits for which MDA status at both visits could be determined, there were 1,390 pairs where the patient did not have MDA at both visits, 825 where MDA was recorded at both visits, 195 where the patients moved from no MDA to MDA, and 144 with movement from MDA to no MDA. These visits are the basis of a complete case analysis.

Table 1 presents the percentage of visits for which information on each of the MDA criteria are missing. The patient global disease activity score is missing approximately 50% of the time, as is the HAQ score, which is only administered once a year. The psoriasis measures and the patient pain score are missing at 16% and 17% of clinic visits, respectively. The remaining criteria are missing less than 10% of the time. In addition, Table 1 presents the percentage of visits at which each criterion was satisfied for both known MDA and known no MDA visits. For example, the patient pain score is hardly ever positive unless MDA is observed, whereas the entheseal points criterion is positive most of the time whatever the MDA status. Therefore, we might expect the patient pain score to be better at discriminating MDA status than entheseal points. The HAQ criterion is satisfied 95% of the time if MDA is observed and only 25% of the time if not. The comparable numbers for the patient global disease activity score criterion are 85% and 5%. The TJC and SJC criteria are satisfied more than 95% of the time when MDA is observed, but are also positive 35% and 52% of the time that MDA is not observed. The psoriasis criterion is positive 76% and 35% of the time when MDA and no MDA, respectively, are observed. Supplementary Figure 1 (available on the *Arthritis Care & Research* web site at http://onlinelibrary.wiley.com/doi/10.1002/acr.22687/abstract) shows histograms of the 8 quantitative variables used to define MDA status along with the thresholds used in the MDA definition.

**Table 1:** Percentage of visits where binary MDA criteria are satisfied, observed, and estimated, and percentage of visits with missing information^a^

	TJC	SJC	PASI+ BSA	PTPAINV	PTPSA	HAQ	ENTH TOT
Missing	4	5	16	17	52	50	6
Complete cases							
Criteria satisfied	67	75	49	28	41	58	94
Satisfied and no MDA	35	52	35	2	5	25	87
Satisfied and MDA	97	96	76	72	85	95	99
Univariate binary model estimates							
Satisfied and no MDA	44	60	35	2	6	27	90
Satisfied and MDA	97	96	72	66	82	96	99
Multivariate binary model estimates							
Satisfied and no MDA	46	62	41	3	6	30	90
Satisfied and MDA	95	94	61	60	79	94	99
Univariate quantitative model estimates							
Satisfied and no MDA	46	63	25	1	2	36	92
Satisfied and MDA	95	95	52	57	76	93	99
Multivariate quantitative model estimates							
Satisfied and no MDA	47	63	32	1	2	38	92
Satisfied and MDA	92	93	43	54	75	92	99

a Values are the percentage. MDA = minimal disease activity; TJC = total joint count; SJC = swollen joint count; PASI = Psoriasis Area and Severity Index; BSA = body surface area; PTPAINV = patient pain visual analog score; PTPSA = patient global activity visual analog score; HAQ = Health Assessment Questionnaire; ENTH TOT = entheseal points.

Also shown in Table 1 are the estimated probabilities of a positive criterion based on the various hidden multistate models. These are broadly consistent with the observed values from the complete case visits, and the differences observed could arise from model misspecification or from an informative observation pattern linked to only using complete case visits and resulting in biased estimation from the complete case analysis.

Table 2 presents the estimated mean time to stay in the MDA and no MDA states, the estimated expected time that a patient starting in the no MDA state will be in the 2 states, and the expected number of episodes in the 2 states over a 10‐year period, as well as the probability of MDA occurring during those 10 years. The latter 3 quantities are also estimated for sustained MDA and, where appropriate, for a transient MDA episode less than 1 year in duration. Results are provided for the complete case, the multivariate binary hidden multistate model, and the multivariate quantitative hidden multistate model, and 95% confidence intervals (95% CIs) are displayed.

**Table 2:** Estimates and 95% confidence intervals for quantities from 3 methods of estimating a 2‐state model for MDA^a^

	Complete case	Multivariate binary	Multivariate quantitative
Mean sojourn time in…			
No MDA	4.06 (3.63–4.53)	3.43 (3.09–3.81)	2.82 (2.56–3.10)
MDA	4.18 (3.65–4.79)	3.82 (3.37–4.33)	3.10 (2.78–3.45)
Expected time over 10 years in…			
No MDA	5.95 (5.69–6.25)	5.69 (5.43–5.95)	5.53 (5.30–5.78)
MDA	4.05 (3.75–4.31)	4.31 (4.05–4.57)	4.47 (4.22–4.70)
MDA episodes lasting <1 year	0.15 (0.12–0.17)	0.18 (0.15–0.21)	0.25 (0.21–0.29)
MDA episodes lasting ≥1 year^b^	3.90 (3.59–4.16)	4.13 (3.88–4.40)	4.22 (3.96–4.45)
First year of MDA	1.26 (1.16–1.34)	1.39 (1.30–1.49)	1.61 (1.52–1.72)
Later years of MDA	2.79 (2.53–3.02)	2.92 (2.70–3.15)	2.86 (2.63–3.07)
Expected number of episodes in…			
No MDA	1.97 (1.85–2.09)	2.13 (2.00–2.26)	2.44 (2.30–2.60)
MDA	1.47 (1.35–1.59)	1.65 (1.53–1.79)	1.96 (1.84–2.12)
MDA lasting <1 year	0.31 (0.26–0.38)	0.38 (0.32–0.45)	0.54 (0.46–0.64)
MDA lasting ≥1 year	1.16 (1.08–1.23)	1.27 (1.20–1.35)	1.42 (1.36–1.50)
Probability of visiting at least once			
MDA	0.92 (0.89–0.94)	0.94 (0.93–0.96)	0.97 (0.96–0.98)
MDA lasting ≥1 year	0.85 (0.82–0.87)	0.88 (0.86–0.91)	0.91 (0.90–0.93)

a Values are the estimate (95% confidence interval). MDA = minimal disease activity.

b Episodes starting between 9–10 years that eventually lasted ≥1 years are counted as “lasting ≥1 year” for this categorization, but the time spent in them is truncated at the 10‐year point.

The mean times to stay in a state are smaller from both hidden multistate models than from the complete case analysis, and the quantitative‐based results are smaller than the binary‐based results. These differences may be related to the fact that the hidden multistate model estimation is based on more frequent observations, and short‐term fluctuations may be suggesting more state changes. There are less dramatic differences in the estimated expected total time, more than 10 years, in the 2 states from the various models, as well as in the expected total time in a sustained MDA state. The expected total time in the first and subsequent years of an MDA episode are also given.

The expected number of episodes in no MDA, MDA, sustained MDA, and transient MDA are also higher when a hidden multistate model is used and it can be seen that more than 2 MDA episodes are not expected over a 10‐year period. The probability of achieving MDA and the probability of sustained MDA at least once in a 10‐year period are similarly estimated to be higher from the hidden multistate models, with the highest coming from the multivariate quantitative results. The estimated probabilities of achieving MDA all exceed 90%, while for sustained MDA the estimates vary from 85% with the complete case analysis to 91% with the multivariate quantitative multistate analysis. It can be seen that there is no overlap between the CIs for both probabilities from the complete case and multivariate quantitative multistate analyses.

Table 2 also illustrates that the estimates from the quantitative and the binary hidden multistate models are more precise (smaller CIs) than the complete case analysis. The SEs from these 3 models for the mean times in the states are 0.17, 0.24, and 0.29, respectively. This is consistent with the greater amount of data used to estimate the hidden multistate models.

**Figure 3:**
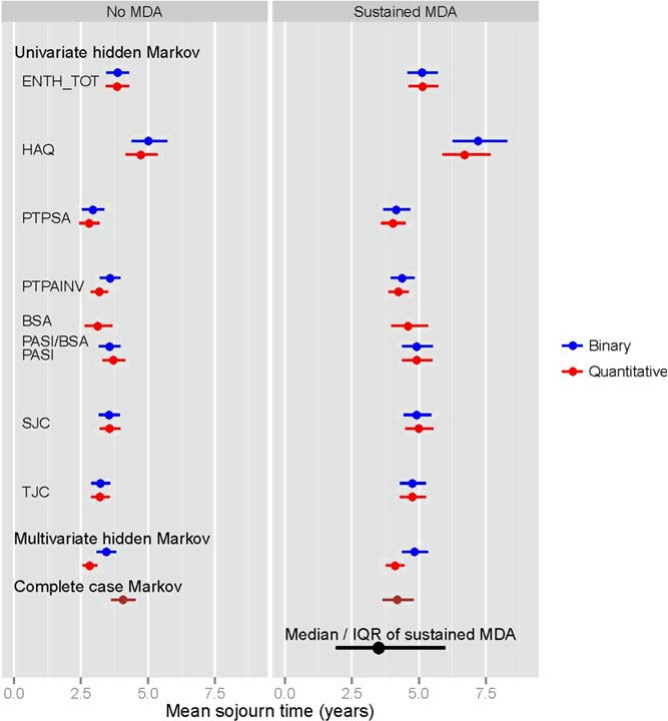
Mean sojourn times with no minimal disease activity (MDA) and MDA, estimated from the hidden multistate and multistate models. ENTH TOT = entheseal points; HAQ = Health Assessment Questionnaire; PTPSA = patient global activity visual analog score; PTPAINV = patient pain visual analog score; BSA = body surface area; PASI = Psoriasis Area and Severity Index; SJC = swollen joint count; TJC = total joint count; IQR = interquartile range.

For illustration purposes, Figure 3 presents the estimated mean sojourn times for the various univariate hidden multistate models and compares them with those arising from the complete case and the 2 multivariate hidden multistate model analyses. For both the TJC and the SJC, slightly shorter mean sojourn times come from the binary variable model in contrast to the results for other univariate models and the multivariate models that have longer times from the binary models. However, the most marked difference arises with the univariate HAQ‐based models, which give much longer sojourn times than the other models. This may arise because there is less fluctuation between HAQ observations (which are only taken once per year) than for the other variables (potentially measured at each visit). Fluctuations will, in a general sense, tend to imply more state changes and this may account for the extreme results based on HAQ only.

## DISCUSSION

Adopting the framework of multistate models, we have shown that investigations of MDA in PsA, which is defined as a composite outcome based on 7 binary criteria, can make use of data from clinic visits at which MDA status can not be determined due to missing data related to these 7 criteria. We have shown that this can provide more precise information but also notable differences in estimates of quantities related to the length of MDA episodes, the cumulative time spent with MDA, and the probability of MDA over a defined time period. Comparable differences are also seen when sustained MDA is examined. The reasons for these differences cannot be unambiguously determined but the possibility of bias, as well as loss of precision, from only using visits at which MDA status is known must be considered. In light of this, conclusions regarding MDA based on complete case analyses should be treated with caution.

Coates et al 1 previously examined MDA in PsA, but as well as requiring 5 of the 7 criteria to be fulfilled, it was also required that MDA must be observed at consecutive visits for a minimum of 12 months in order to focus on sustained MDA. In our current data set, which updates that of Coates et al and is based on complete case data, 229 of 619 patients (37%) achieved this, and the median duration of such episodes was 42 months (3.5 years), which is greater than the median of 28 months presented by Coates et al based on earlier data on 344 patients. While there may be other reasons for this difference, the difference is at least partially explained simply on the basis of followup times, as the length of MDA episodes will be censored at the last observation time. For these episodes in our data, which begin prior to 2008 and is the cut off for the data of Coates et al, the mean duration is 76 months (6.3 years), reflecting the additional followup of the patients considered by Coates et al. For MDA episodes in our data beginning after 2007, the mean duration is 27 months (2.3 years). Therefore, estimation of the length of MDA episodes in this manner is problematic and the estimated mean durations arising from a 2‐state model should be preferred as these are valid estimates not influenced by followup times.

Our 2‐state model can also be extended to allow the transition rates to depend on calendar time and therefore to address specifically whether MDA has been seen more frequently in recent years. In doing so, we estimate, using our quantitative hidden multistate model, that the rate of transition to MDA is estimated to be increased by a factor of 1.2 (95% CI 1.0–1.5) in the years 2007–2009 and by a factor of 1.3 (95% CI 1.1–1.7) in the years 2010+, compared with the period prior to 2007. There is also some suggestive evidence that the rate of transition back to no MDA may have also increased. From the same model, the rate of transition out of MDA is estimated to be increased by factors of 1.3 (95% CI 1.0–1.7) and 1.1 (95% CI 0.9–1.5) for the periods 2007–2009 and 2010+, respectively.

As was done to examine this dependence of transition rates on calendar time, it is relatively straightforward to generalize the multistate model to allow the transition rates to depend on other explanatory variables so that predictors of MDA can be investigated. However, our aim in this study has been to highlight some potentially important issues in the use and modelling of MDA data.

## AUTHOR CONTRIBUTIONS

All authors were involved in drafting the article or revising it critically for important intellectual content, and all authors approved the final version to be submitted for publication. Dr. Farewell had full access to all of the data in the study and takes responsibility for the integrity of the data and the accuracy of the data analysis.

### Study conception and design

Jackson, Su, Farewell.

### Acquisition of data

Gladman.

### Analysis and interpretation of data

Jackson, Su, Gladman, Farewell.

## Supporting information

SUPPLEMENTARY FIGURE 1: On Modelling Minimal Disease Activity
