# Supplementary material for: On Modelling Minimal Disease Activity
Source: Arthritis Care Res (Hoboken). 2016 Feb 23;68(3):388–93. doi: 10.1002/acr.22687 (PMC4949508; doi:10.1002/acr.22687)
Supplement: Supplementary file 1 — SUPPLEMENTARY FIGURE 1: On Modelling Minimal Disease Activity [file ACR-68-388-s001.docx]

SUPPLEMENTARY FIGURE 1: On Modelling Minimal Disease Activity


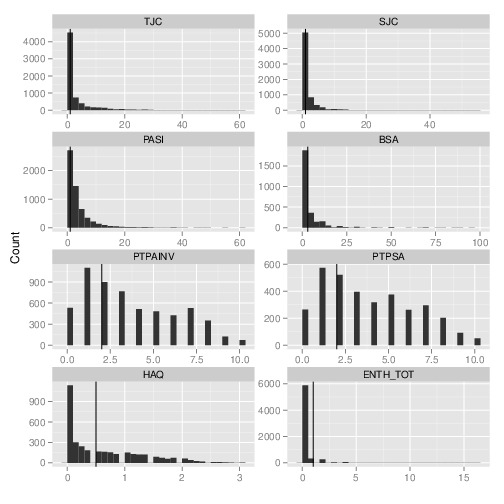


Histograms of variables used in MDA defining criteria. Thresholds used in the MDA definition indicated as vertical lines.

Abbreviations: TJC (Total joint count); SJC (Swollen joint count); PASI (Psoriasis Area and Severity Index); BSA (Body Surface Area); PTPAINV (Patient pain visual analogue score); PTPSA (Patient global activity visual analogue score); HAQ (Health Assessment Questionnaire); ENTH_TOT (Entheseal points)
